# Exosomes derived from umbilical cord-mesenchymal stem cells inhibit the NF-κB/MAPK signaling pathway and reduce the inflammatory response to promote recovery from spinal cord injury

**DOI:** 10.1186/s13018-024-04651-w

**Published:** 2024-03-16

**Authors:** Zhiwei Luan, Jingsong Liu, Mi Li, Yangyang Wang, Yansong Wang

**Affiliations:** 1 Department of Orthopedic surgery, The First Affiliated Hospital of Harbin Medical University, Harbin, China; 2 NHC Key Laboratory of Cell Transplantation, Harbin Medical University, Harbin, China; 3 The Key Laboratory of Myocardial Ischemia, Chinese Ministry of Education, Harbin, China; 4 Heilongjiang Provincial Key Laboratory of Hard Tissue Development and Regeneration, Harbin Medical University, Harbin, China

**Keywords:** Spinal cord injury, Transplanted mesenchymal stem cells, Exosomes, Inflammation, Sequencing

## Abstract

Spinal cord injury (SCI) is a serious traumatic disease of the central nervous system and leads to incomplete or complete loss of the body’s autonomous motor and sensory functions, seriously endangering human health. Recently, exosomes have been proposed as important substances in cell-to-cell interactions. Mesenchymal stem cell (MSC)-derived exosomes exert good therapeutic effects and play a crucial role in neurological damage repair. However, the detailed mechanisms underlying their effects remain unknown. Herein, we found that compared to SCI rats, those subjected to umbilical cord MSC (UC-MSC)-derived exosomes injection showed an improved motor ability. Nevertheless, the transcriptome of BV2 microglia in different treatment groups indicated that the action pathway of exosomes might be the NF-κB/MAPK pathway. Additionally, exosomes from UC-MSCs could inhibit P38, JNK, ERK, and P65 phosphorylation in BV2 microglia and SCI rat tissues. Moreover, exosomes could inhibit apoptosis and inflammatory reaction and reactive oxygen species (ROS) production of BV2 microglia in vitro and in vivo. In conclusion, UC-MSCs-derived exosomes might protect SCI in rats by inhibiting inflammatory response via the NF-κB/MAPK signaling pathway, representing novel treatment targets or approaches for SCI.

## Introduction

Spinal cord injury (SCI) is an extremely serious nervous system disorder that permanently impairs motor and sensory abilities, along with various pathological alterations, including tissue hemorrhage, edema, and local inflammation [1, 2]. Around 2.5 million people worldwide suffer from traumatic SCI, with over 130,000 new cases reported annually [3]. SCI is divided into primary and secondary. Falls, traffic accidents, exercise, and violence can induce primary SCI [4, 5]. Secondary SCI initiates shortly after the primary injury and can endure for several weeks or months, spreading the injury from the affected area to the surrounding tissue. Secondary SCI can also lead to inflammation, edema, necrotic cell death, and vascular damage. However, no effective treatments are available for SCI [6, 7].

As a kind of stem cells, mesenchymal stem cells (MSCs) have self-renewal ability and multiple differentiation potentials. They come from various sources and have low immunogenicity, making them ideal grafts for tissue engineering repair. In recent years, the strong proliferative and differential capacities of mesenchymal stem cells (MSCs) have led to new progress in SCI treatment [8]. As recognized anti-inflammatory response barriers, MSCs are currently the best SCI cell transplantation therapy choice. Due to the therapeutic effects of MSC transplantation for SCI, these cells have entered the clinical stage of SCI treatment [9, 10].

Umbilical cord mesenchymal stem cells (UC-MSCs) exist in the umbilical cord tissue connected to the fetus after delivery. They are widely used as seed cells for tissue regeneration and repair due to their low ethical issues, multi-directional differentiation potential, immune regulation, and biological anti-inflammatory effects [11, 12]. The differentiation potential of UC-MSCs into diverse cell types, such as bone, cartilage, and myocardium, has been evidenced by research under in vitro and in vivo induction conditions [13, 14]. UC-MSCs rarely express human leucocyte antigen I (HLA-I), human leucocyte antigen DR (HLA-DR), and co-stimulatory molecules CD40 and CD80, thereby presenting low immunogenicity and good post-transplant receptor tolerance [15, 16]. Therefore, UC-MSC transplantation might become the most promising method for treating SCI due to its advantages of convenient extraction of primary cells, less contamination, and low immunogenicity [17, 18].

Exosomes originate from intracellular vesicles and are small membrane vesicles with a diameter ranging from approximately 30 to 200 nm. These vesicles have diverse proteins, lipids, and RNAs. Cells from all living systems can release these vesicles into the cerebrospinal fluid, blood, and extracellular fluid [19]. After SCI occurrence, how to effectively improve its pathological and physiological changes has become a key point in the treatment and prognosis of rehabilitation effects. Compared with simple stem cell transplantation, the application of an exosome cell-free diagnosis and treatment scheme will not cause tumor risk, immune reaction, infection, and other complications caused by living cell transplantation [20]. Moreover, due to the nano diameter of exosomes, they are not easily captured and degraded by tissues such as the lungs and liver after use, and can smoothly pass through the tissue barrier, thus concentrating in the lesion area [21]. Therefore, exosomes have certain feasibility and theoretical basis for SCI treatment. Until now, many in vivo and in vitro studies have demonstrated that exosomes play a good role in SCI treatment [22], promoting nerve regeneration and angiogenesis and reducing inflammatory reactions, cell apoptosis, and scar tissue [23].

Herein, we explored the finctions of UC-MSC-derived exosomes in SCI. Combined with the transcriptome analysis, we found that these exomes might alleviate inflammatory responses in cells and tissues via the NF-κB/MAPK pathway. Overall, we provided new insights and assistance for future SCI treatments.

## Materials and methods

### Cell culture and treatment

BV2 microglia were purchased from Procell Biotechnology Co., Ltd. (Wuhan, Hubei, China). Cells were placed in α-MEM medium containing FBS (10%) and penicillin and streptomycin (1%), then cultured at 37 ℃ and 5% CO_2_. BV2 microglia were stimulated with LPS for 12 h, and ATP (5 mM Sigma, USA) was added for 30 min. The concentration of UC-MSCs added was 20 µ g/mL.

UC-MSCs were provided by Procell Biotechnology Co., Ltd. (Wuhan, Hubei, China). Cells were inoculated in a 1:2 ratio and subcultured in a T25 culture flask. Then, the fresh complete culture medium was added to 5 mL and placed in a cell culture incubator at 37 ℃, 5% CO_2_, and saturated humidity for static cultivation. When the cell fusion degree was about 60–70%, the original FBS-containing medium was removed and replaced by a fresh serum-free medium. Cell culture continued for about three days. The cell supernatant was collected when the cell fusion degree was about 80–95%.

### Identification of exosomes

As previously described [24], exosomes were extracted from the supernatant of UC-MSCs using ultracentrifugation. The morphology of extracted exosomes was analyzed using transmission electron microscopy (TEM), and the diameter and size were evaluated using nanoparticle tracking analysis (NTA). Then CD9, CD63, and CD81 expression were examined Western blot.

### Reactive oxygen species (ROS)

BV2 cells were digested, counted, and placed in a 24-well plate, ensuring the number of cells was equal in each well. The probe was diluted with medium (1:1000) to 10 µM. After removing the old medium and washing the cells using PBS, 500 µL of the probe solution was added to each well and incubated for 20 min in an incubator at 37 ℃. The probe’s working solution was discarded, washed gently with PBS thrice, and 400 µL of the medium was added to each well. Finally, a fluorescence microscope was used for observation.

### RNA sequencing (RNA-seq)

Total RNA extraction was conducted after cell digestion in QIAZOL (Qiagen). A TruSeq Stranded mRNA Sample Prep Kit (Illumina) was used to prepare the library. The 75-base single-end mode was applied for the sequencing on an Illumina HiSeq 2500 platform. Base calling was performed using Illumina Casava 1.8.2. The Integrated Differential Expression and Pathway (iDEP) analysis software (Version 94) was used for bioinformatics analysis.

### Immunofluorescence

For BV2 microglia subcultures, cells were inoculated into the culture dish with the treated cover glass. After cells had grown into a monolayer, the cover glass was removed and soaked with PBS thrice (three min each). Then, the slide was fixed with paraformaldehyde for 15 min and soaked thrice with PBS. Next, the slices were permeated for 20 min and soaked thrice with PBS (three min each) at room temperature (RT). The slide was blocked for 30 min using normal goat serum at RT. Then, the slide was incubated with diluted primary antibodies at 4 ℃. On the second day, samples were incubated with the diluted fluorescent secondary antibody at 37 ℃ for 1 h. Finally, one drop of sealing agent was added, and samples were observed with a fluorescence microscope.

### RT-PCR and Western blot

Quantitative RT-PCR and Western blot analyses were performed as previously described [25, 26]. The primers for PCR are shown in Table 1.

**Table 1:** Primers for qRT-PCR analysis

Gene	Forward Premier	Reverse Premier
INOS	CCCTTCAATGGTTGGTACATGG	ACATTGATCTCCGTGACAGCC
TNF-α	CTCAAGCCCTGGTATGAGCC	GGCTGGGTAGAGAACGGATG
IL-1β	AGCTTCAGGAAGGCAGTGTC	TCAGACAGCACGAGGCATTT
IL-6	AGAGACTTCCAGCCAGTTGC	AGTCTCCTCTCCGGACTTGT

### Animal experiments

Fifteen Sprague Dawley rats were divided into three groups: Sham, SCI, and SCI + exosomes. In the SCI group, 0.5 mL PBS was injected into the tail vein of rats, while the same volume of UC-MSCs (200 µg/mL) was injected into the tail vein of rats in the SCI + exosomes group.

Rats were anesthetized by intraperitoneal injection of 3.6% chloral hydrate at 1 mL/100 g. After 3–5 min, rats experienced unstable standing and weak or disappeared pain responses. Considering T8 and T9 as the center, a midline incision (1-1.5 cm length) was made on the back. The fascia and muscles were separated layer by layer, the spinal spinous processes of T8 to T10 segments were exposed, and the muscles on the spinal vertebrae of T8 and T10 segments were passively separated. The bone forceps were bitten to remove the spinous processes and lamina of T8 and T10 segments, and the T10 spinal cord was exposed. A 10 g metal rod was selected to fall freely at a distance of 30 mm and hit the T10 nerve segment. Tail swinging and hind limb retraction flutter marked the successful SCI. After the blow, the muscles, fascia, and skin were disinfected using sterile surgical suture needles and threads, followed by intraperitoneal injection of 2 mL glucose solution and intramuscular injection of 1 mL (8 U/mL) penicillin. Within one week after surgery, the wound was disinfected, and 1 mL (8 U/mL) of penicillin was injected intramuscularly. Experimental animals received help to urinate thrice a day until they could urinate autonomously. Two weeks after surgery, spinal cord tissue was taken from rats for subsequent experiments.

### Behavioral evaluation

Experimental rats were evaluated with Basso Beattie Bresnahan (BBB) 3, 7, 14, 21, and 28 days after surgery. The bladder was evacuated before observation to avoid affecting the rats’ activity. Then, animals were placed on a flat and unsmooth experimental platform and observed for four min. Behavioral scores were conducted on the animal’s body positioning and hind limb function. The raters are non-experimental personnel familiar with the scoring criteria, and the average value was taken based on three scores.

### Enzyme-linked immunosorbent assay (ELISA)

Rat spinal cord samples were collected, placed in 0.25 mL of extraction buffer, and ground. The homogenate was centrifuged at 20,000 g at 4 ℃ for 0.5 h. The supernatant was equally divided and stored at -80 ℃ for cytokine detection. INOS, IL-6, IL-1β, TNF-α and were quantitatively analyzed using ELISA kits.

### Hematoxylin and Eosin (HE) and Nissl stainings

We take pathological sections from two weeks after SCI for experimentation. For HE staining, sections were placed into a hematoxylin aqueous solution for staining for a few minutes, followed by acid and ammonia water for color separation for a few seconds. After one hour of rinsing using running water and 10 min of dehydration using 70 and 90% alcohol solutions, sections were stained for three min using eosin. After dehydration using 100% alcohol and transparentizing using xylene, stained sections were sealed and observed under a microscope.

For Nissl staining, sections were stained with Nissl staining solution at 56 ℃ for 10–15 min, directly differentiated with 95% alcohol differentiation solution for a few seconds, and quickly washed with water. Finally, samples were dehydrated with anhydrous alcohol twice for two minutes, transparentized with xylene twice for five minutes, and sealed with neutral resin.

### Immunohistochemistry

Two weeks after establishing the SCI rat model, fresh spinal cord tissues were collected and fixed with 4% paraformaldehyde.Then, tissues were dehydrated, embedded in paraffin, and sliced to a thickness of 3 μm using a paraffin slicer. After dewaxing and antigen repair, the primary antibody was added to the slices overnight, and the second antibody was applied for one hour the next day. Finally, the staining was observed under a microscope.

### Statistical analysis

Data were analyzed using SPSS statistical software. Results are presented as means ± standard deviations. One-way analysis of variance (ANOVA) was conducted to analyze the data. *p* < 0.05 was considered statistically significant.

## Results

### Identification of UC-MSC-derived exosomes

**Fig. 1:**
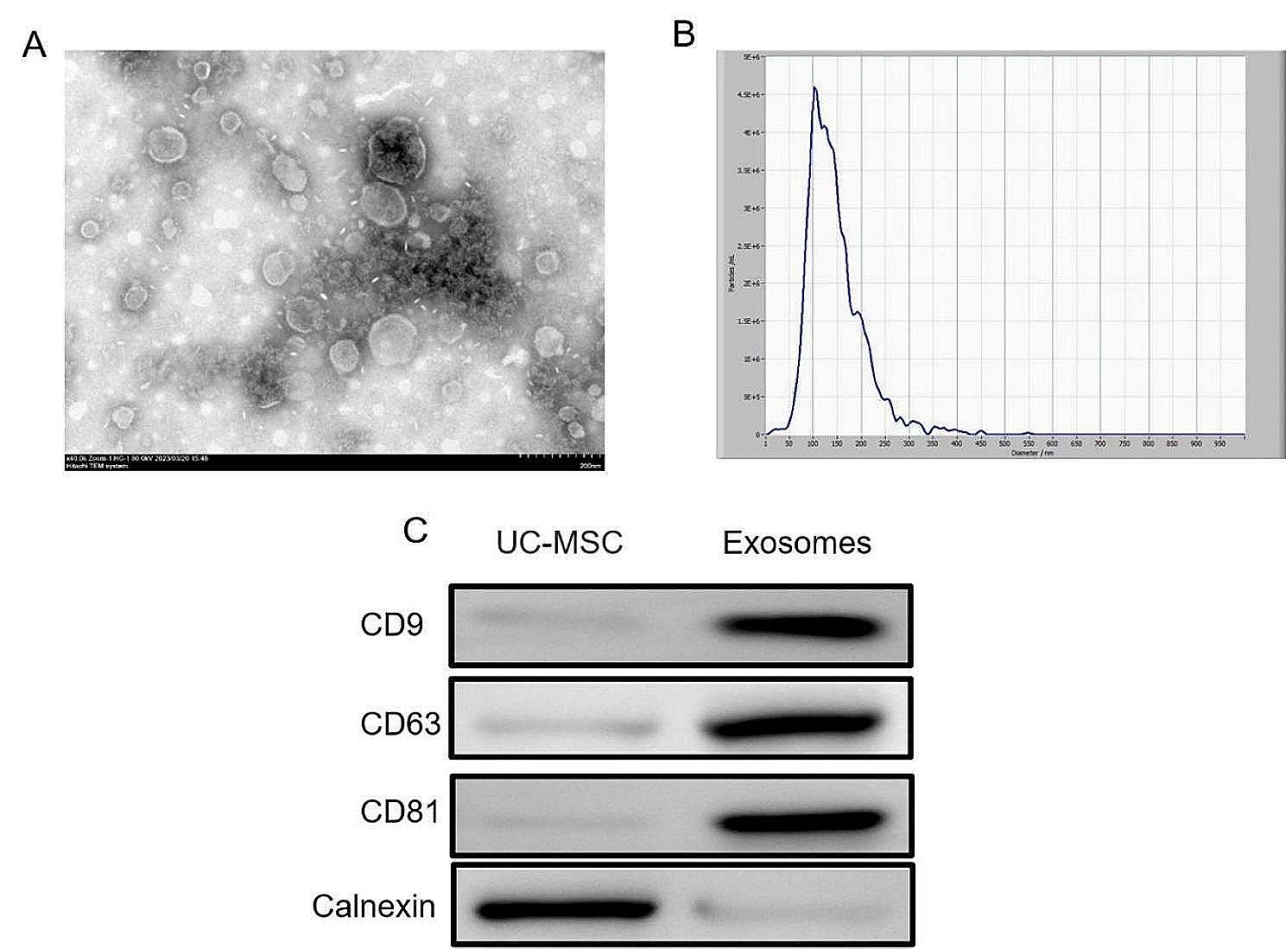
Identification of exosomes derived from UC-MSCs. (**A**) Representative images showing the TEM observation of exosomes’ discoid vesicular structure. (**B**) Exosomes diameter by NTA. (**C**) Representative blots showing CD81, CD63, CD9 and Calnexin levels. Scale bar = 200 nm

First, we extracted exosomes using ultracentrifugation and observed their disc-shaped vesicular structure using TEM (Fig. 1A). The NTA showed that the diameter of the exosomes was mostly between 30 and 200 nm (Fig. 1B). We conducted Western blot analysis for specific markers of extracellular vesicles, and successfully detected CD9, CD63, CD81 and Calnexin (Fig. 1C).

### Sequencing analysis of BV2 microglia

**Fig. 2:**
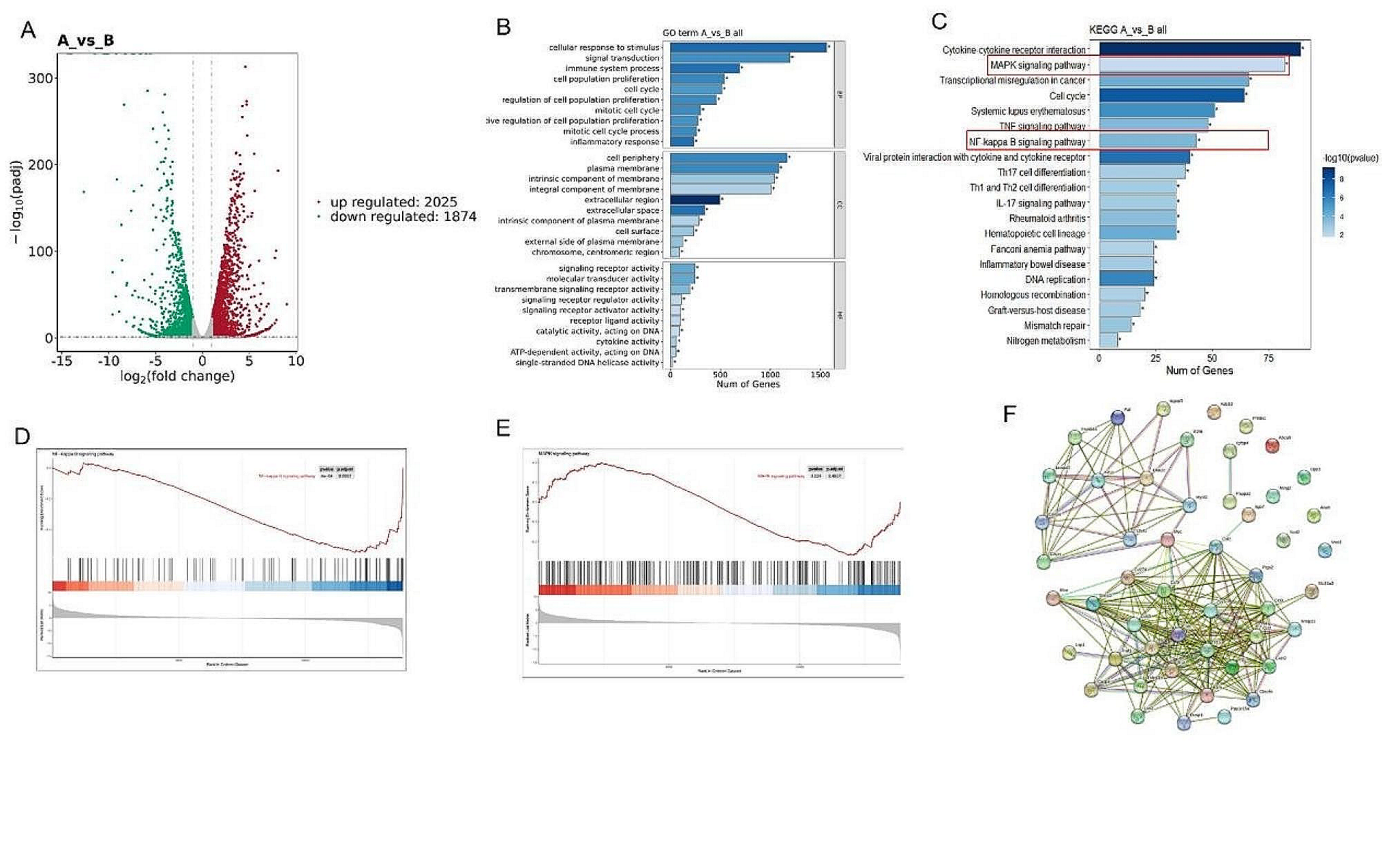
Bioinformatics analysis of BV2 microglia after sequencing. (**A**) Representative Volcano plot showing the differential genes. Downregulated genes are represented as green dots and upregulated genes as red dots. (**B**) GO and (**C**) KEGG enrichment analysis of differential genes. GSEA of (**D**) NF-κB and (**E**) MAPK pathways. (**F**) PPI networks of differentially treated BV2 microglia

To elucidate the underlying mechanisms of UC-MSC-derived exosomes on BV2 microglia, we conducted RNA-seq on differentially treated BV2 microglia. Group A was treated with LPS + ATP + Exo, and Group B was treated with LPS + ATP. We sequenced three samples from each group and performed different bioinformatics analyses. Group A presented 3899 differential genes compared to Group B, 1874 downregulated and 2025 upregulated (Fig. 2A). Then, we performed KEGG and GO enrichment analyses using these differential genes. These genes were significantly enriched for inflammatory response (Fig. 2B). Based on the KEGG enrichment analysis, we selected the NF-κB and MAPK pathways for further analysis (Fig. 2C). Next, the Gene Set Enrichment Analysis (GSEA) showed that the peak of the curve was in the lower part, indicating that NF-κB and MAPK pathways were active in group B (Fig. 2D and E). Finally, we constructed a protein-protein interaction (PPI) network and found that tumor necrosis factor (TNF) and IL1β were strongly associated in both groups (Fig. 2F).

### UC-MSC-derived exosomes inhibit the inflammatory response and ROS production in BV2 microglia

**Fig. 3:**
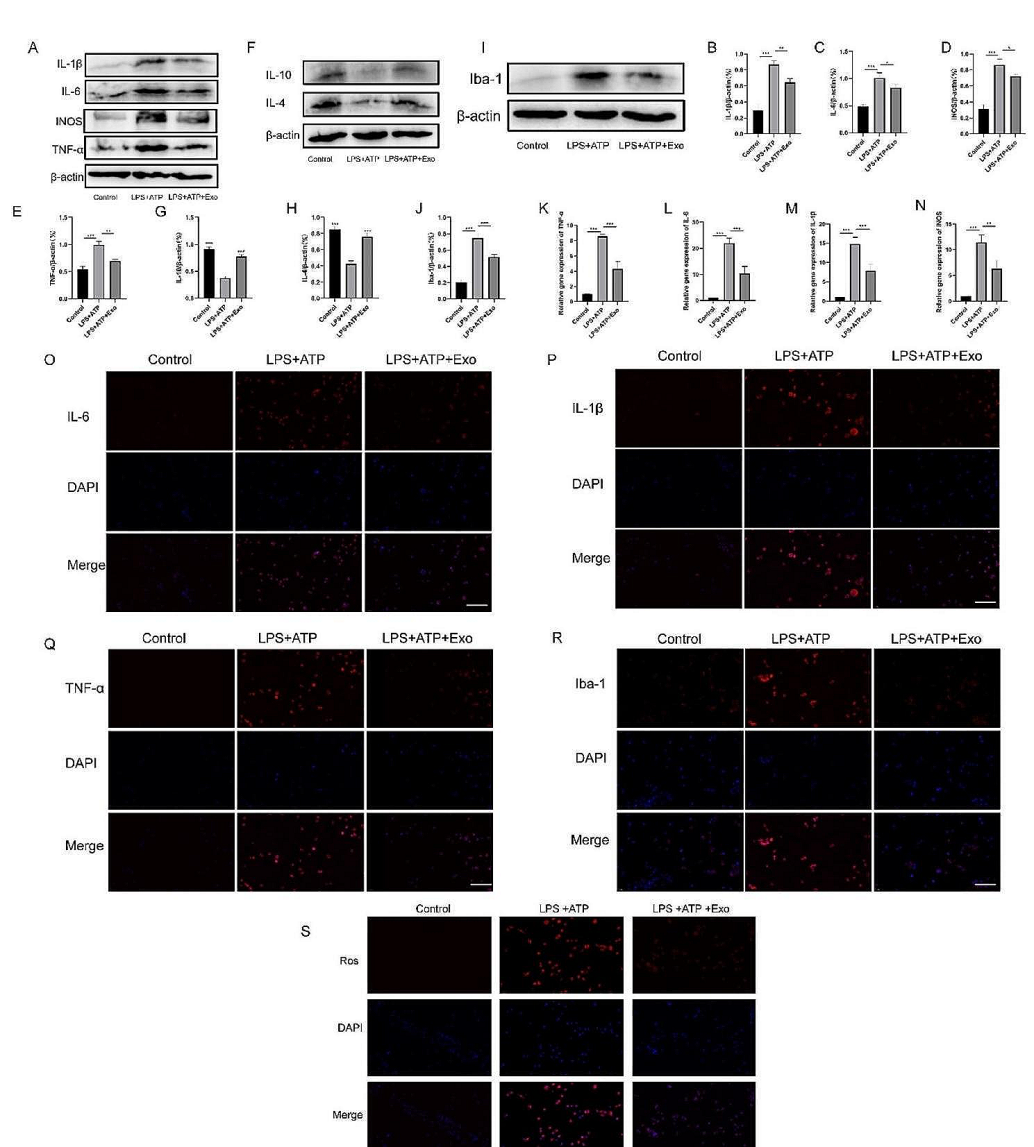
UC-MSC-derived exosomes inhibited the inflammatory response of BV2 microglia and ROS production. (**A**) Representative blots showing the levels of inflammatory factors in exosomes-treated BV2 microglia. (**B-E**) Representative graphs showing the relative expression of IL-1β (**B**), IL-6 (**C**), INOS (**D**) and TNF-α (**E**). (**F**) Representative blots showing the levels of Anti-inflammatory factors in exosomes-treated BV2 microglia. (**G-H**) Representative graphs showing the levels of IL-10 (**G**) and IL-4 (**H**) in BV2 microglia. (**I-J**) Exosomes inhibit the expression of Iba-1 in microglia. (**K-N**) Representative graphs showing the levels of TNF-α (**K**), IL-6 (**L**), IL-1β (**M**), and INOS (**N**) in BV2 microglia. (**O-R**) Representative images showing the levels of IL-6 (O), IL-1β (P), TNF-α (Q), and Iba-1 (R) by immunofluorescence. (S) Exosomes inhibited ROS production in cells. Scale bar = 50 μm. *p < 0.05, **p < 0.01, ***p < 0.001

To explore the effects of exosomes derived from UC-MSCs on BV2 microglia, we performed Western blot and immunofluorescence analysis on Iba-1, the signature of microglia. LPS + ATP upregulated Iba-1, while exosomes downregulated (Fig. 3I, J, R). Inflammation is an essential stage during SCI. To explore the effects of UC-MSC-derived exosomes on the cellular inflammatory response, we performed Western blot on BV2 microglia (Fig. 3A). IL-1β, IL-6, INOS, TNF-α and levels were significantly enhanced after LPS + ATP stimulation but were suppressed by exosomes (Fig. 3B-E). The results of PCR experiments and immunofluorescence are consistent with Western blot results (Fig. 3K-Q). The changes in anti-inflammatory factors are opposite to those in pro-inflammatory factors (Fig. 3F-H). We also detected ROS levels in cells since they can aggravate inflammation to a certain extent. LPS + ATP increased ROS levels, while exosomes reduced LPS + ATP-induced ROS production (Fig. 3S).

### UC-MSC-derived exosomes inhibit BV2 microglia apoptosis

**Fig. 4:**
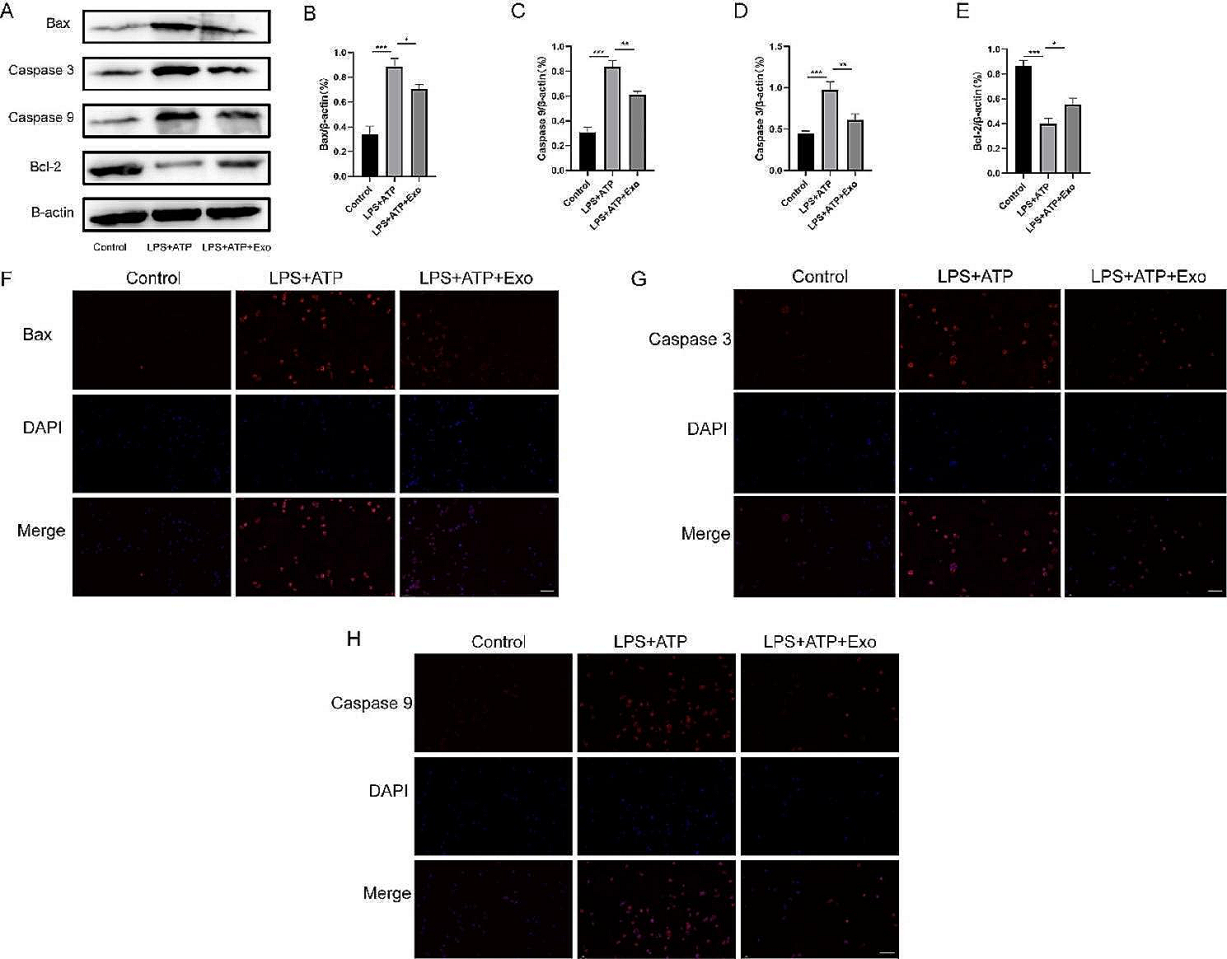
UC-MSC-derived exosomes inhibited the apoptosis of BV2 microglia. (**A**) Effects of exosomes on the apoptosis of BV2 microglia by Western blot. (**B**) Bax, (**C**) Caspase 9, (**D**) Caspase 3, and (**E**) Bcl-2 relative level to β-actin in BV2 microglia. Levels of apoptotic factors (**F**) Bax, (**G**) Caspase 3, and (**H**) Caspase 9 by immunofluorescence. Scale bar = 50 μm. *p < 0.05, **p < 0.01, ***p < 0.001

Apoptosis influences SCI. Hence, we explored the effects of UC-MSC-derived exosomes on the apoptosis of BV2 microglia. In the Western blot assay, Bax, Caspase 9 and Caspase 3 were significantly enhanced, while Bcl-2 was downregulated in LPS + ATP-treated cells (Fig. 4A-E). After exosome treatment, subsequent immunofluorescence tests also showed dramatically reduced apoptosis of cells in the LPS + ATP group, suggesting that exosomes inhibited apoptosis (Fig. 4F-H).

### UC-MSC-derived exosomes inhibit the NF-κB and MAPK pathways in BV2 microglia

**Fig. 5:**
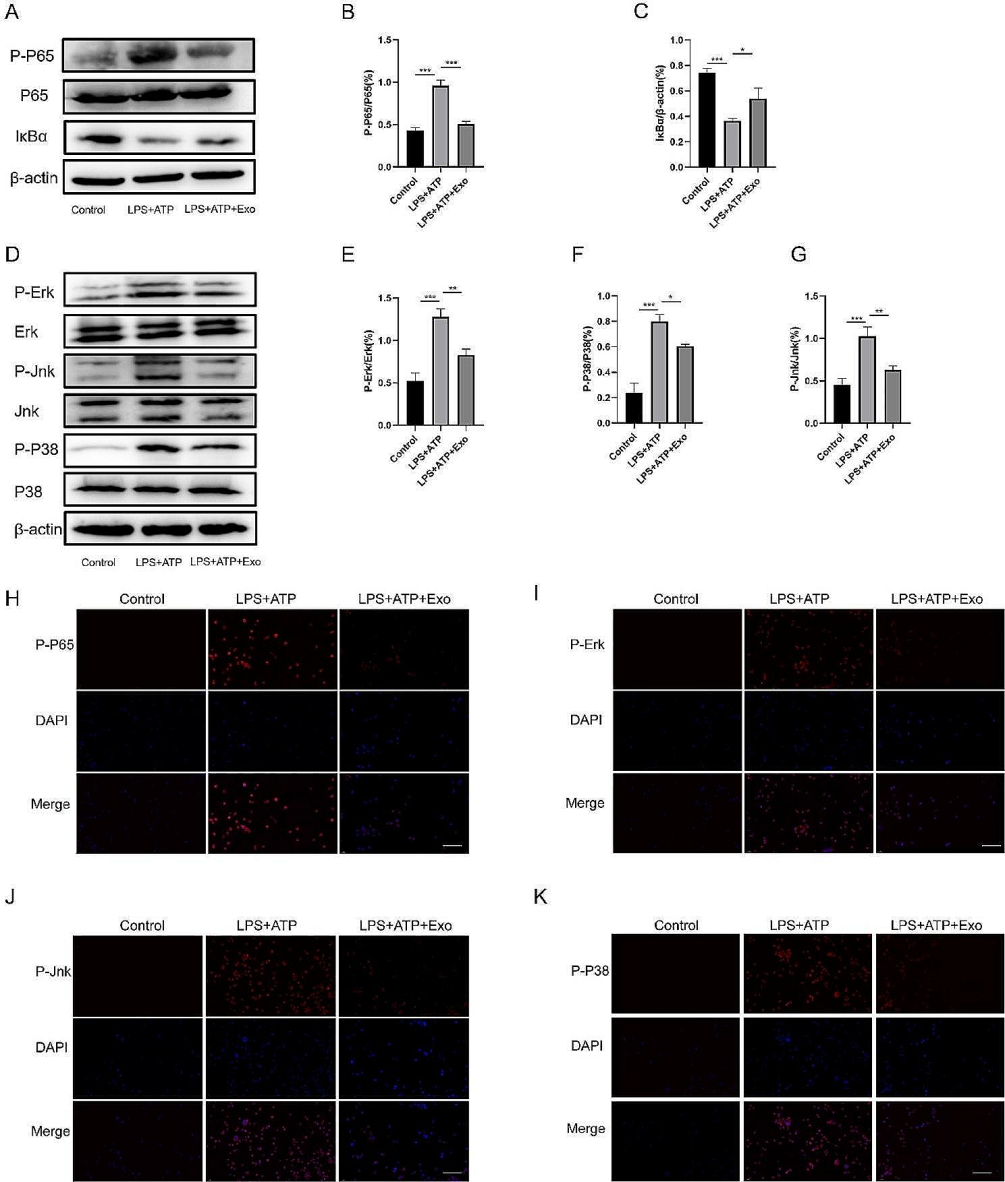
UC-MSC-derived exosomes inhibited the MAPK/NF-κB pathway in BV2 microglia. (**A**) Representative blots showing the levels of P-P65 and IκBα in cells after exosome treatment. Relative levels of (**B**) P-P65 to P65 (**C**) and IκBα to β-actin. (**D**) Representative blots showing the P-P38, P-Jnk, and P-Erk levels in cells after exosome treatment. (**E-G**) Representative graphs showing the relative expression of P-Erk (**E**), P-P38 (**F**), and P-Jnk (**G**). (**H-K**) Representative images showing the levels of P-P65 (**H**), P-Erk (**I**), P-Jnk (**J**), and P-P38 (**K**) by immunofluorescence. Scale bar = 50 μm. *p < 0.05, **p < 0.01, ***p < 0.001

Next, we explored the underlying mechanisms of how UC-MSC-derived exosomes affect SCI. We observed significant enrichments of differential genes in the NF-κB and MAPK pathways, and the GSEA showed that they were actively expressed in the LPS + ATP group (Fig. 2C-E). Thus, we performed Western blot and immunofluorescence analyses of microglia to test our hypothesis. For the NF-κB pathway, LPS + ATP promoted P65 phosphorylation but inhibited IκBα expression (Fig. 5A-C). The exosomes reduced P-P65 protein levels and promoted the IκBα expression.This is consistent with the results of immunofluorescence (Fig. 5H). For the MAPK pathway, exosomes inhibited LPS + ATP-induced ERK, P38 and JNK phosphorylation (Fig. 5D-G). Immunofluorescence also indicated that exosomes inhibited the MAPK pathway in BV2 microglia (Fig. 5I-K).

### UC-MSC-derived exosomes enhance SCI recovery in rats

**Fig. 6:**
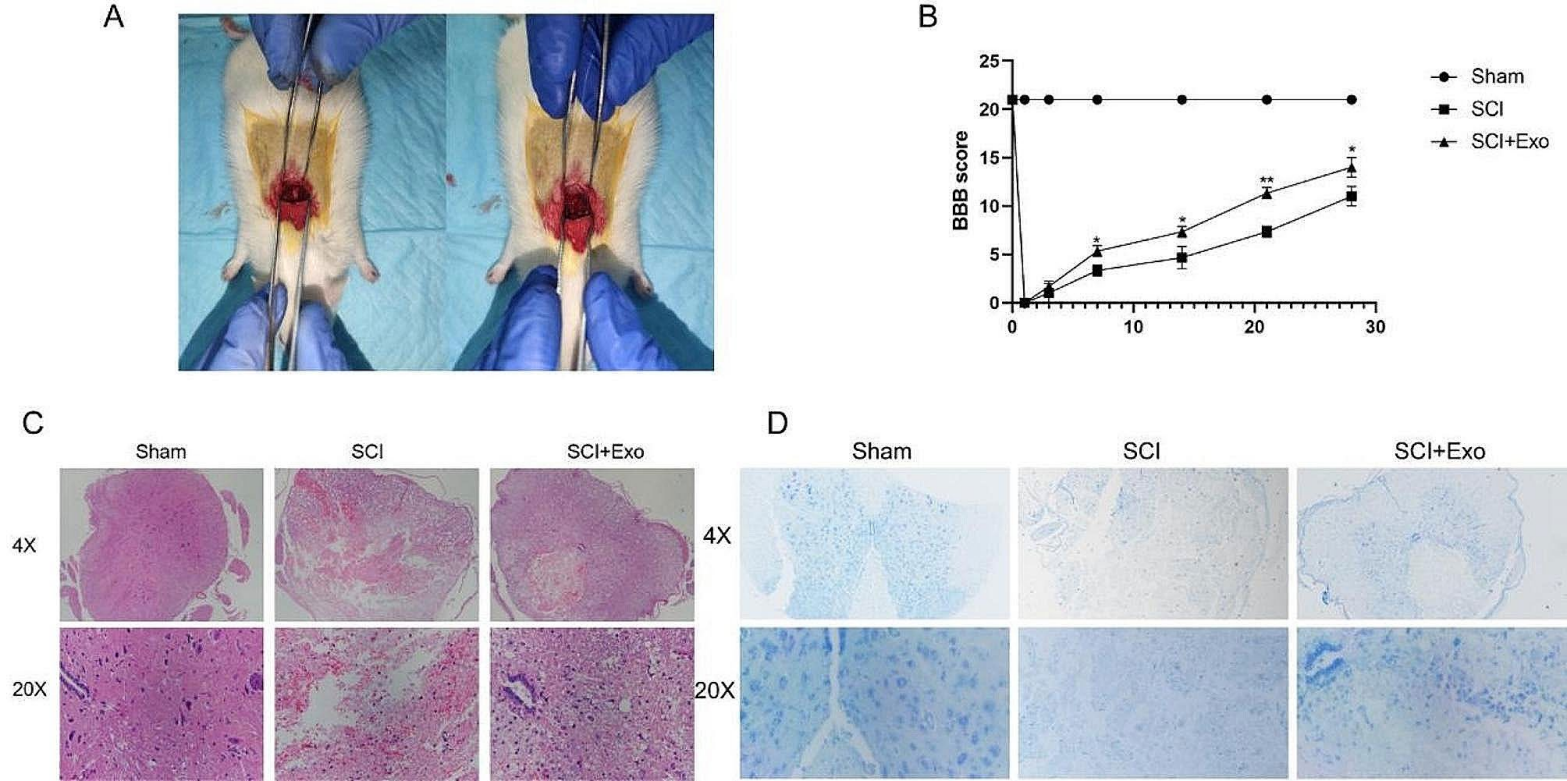
UC-MSC-derived exosomes can alleviate SCI in rats. (**A**) Images of rat modeling surgery. (**B**) BBB score of rats 1, 3, 7, 14, 21, and 28 days after SCI. (**C**) Representative images showing the severity of spinal cord damage in SCI rats by HE staining. The spinal cord tissue of the exosome group was improved. (**D**) Nissl staining showed that the number of neurons in the spinal cord tissue of rats in the exosomes group was much higher than that in the SCI group

Further, to verify the therapeutic effects of UC-MSC-derived exosomes on SCI rats, we evaluated the protective effect of exosomes on acute SCI using the SCI rat model. To determine whether exosomes improved hind limb motor function in SCI rats, we conducted a behavioral study in which the recovery of hind limb function was evaluated using the BBB motor rating scale (Fig. 6B). In the first three days after modeling, all rats completely lost the ability to move their hind limbs. Subsequently, hind limb motor function recovery presented varying degrees at 3–28 days after injury, and a gradually increased BBB score was observed. A significantly high BBB score was observed seven days after surgery in the rats in the exosome group compared to the SCI group. Specifically, rats in the SCI group generally showed extended hind limbs without weight support and could only perform simple joint movements without weight bearing or walking. In contrast, those in the exosome group could frequently walk with plantar weight support and occasionally showed coordinated fore-hind limb movement.

To understand the pathological changes in motor function recovery after SCI, we used HE staining to analyze histomorphology changes caused by SCI (Fig. 6C). The SCI group had the worst spinal anatomy, and the main manifestations were the loss of morphological integrity of the spinal cord and a large number of inflammatory cells infiltrating the spinal cord. The lesion area and capsule volume were reduced in the exosome group, and a slight inflammatory cell infiltration was detected. Overall, the treatment with extracellular vesicles significantly improved spinal cord continuity and reduced lesion cavities, consistent with the BBB score. The Nissl staining showed that the SCI rats had dramatically reduced spinal cord neurons (*p* < 0.05), which might cause motor dysfunction after SCI. Meanwhile, the exosomes group had significantly increased spinal cord neurons (Fig. 6D).

### UC-MSC-derived exosomes inhibit inflammation in SCI rats

**Fig. 7:**
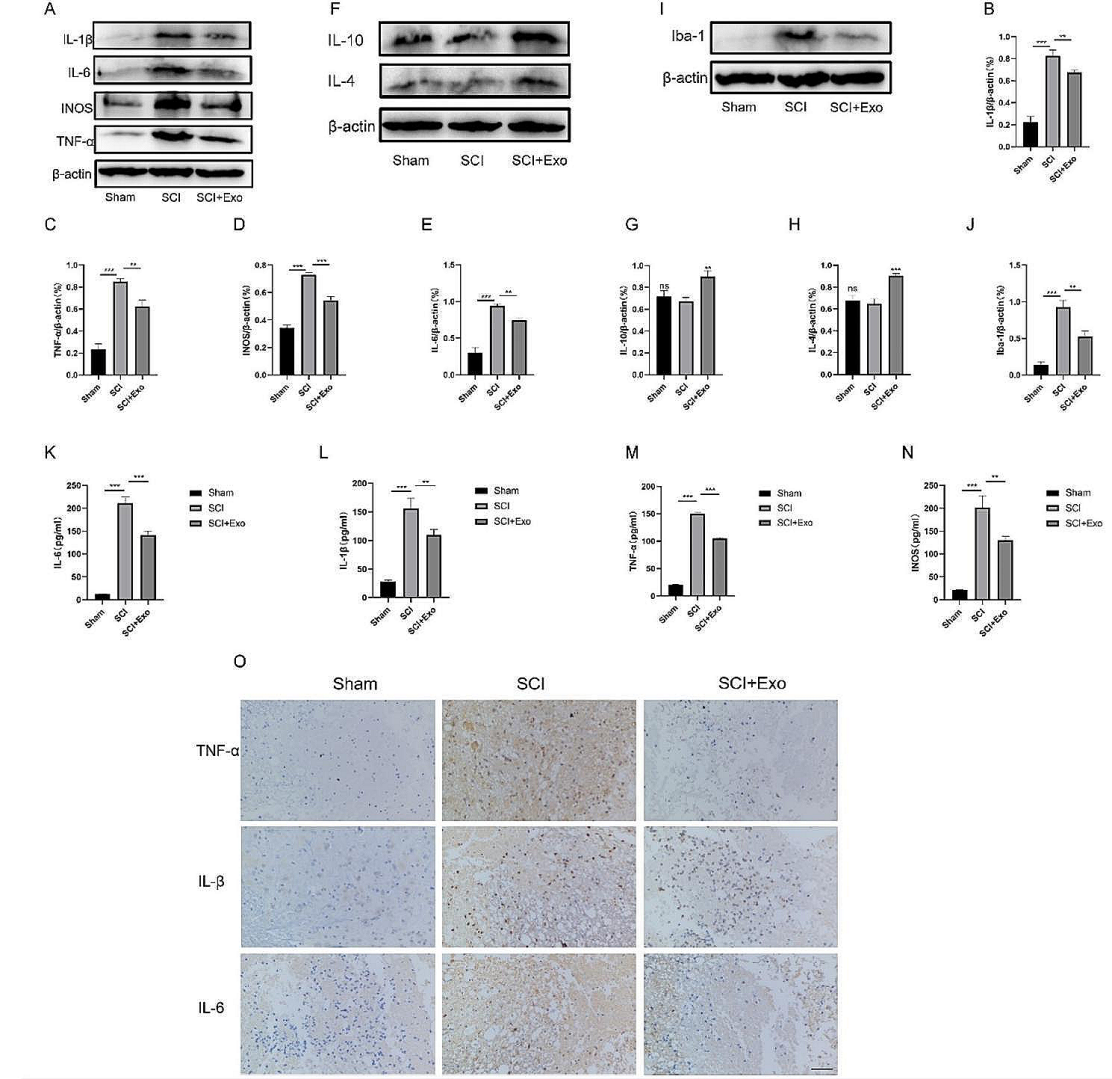
UC-MSC-derived exosomes inhibit the inflammatory response of rat spinal cord tissue. (**A**) Effects of exosomes on spinal cord inflammation in rats by Western blot. (**B-E**) Representative graphs showing the relative levels of IL-1β (**B**), TNF-α (**C**), INOS (**D**) and IL-6 (**E**) in spinal cord tissue to β-actin. (**F**) Effects of exosomes on spinal cord anti-inflammation factors in rats by Western blot. (**G-H**) Representative graphs showing the relative levels of IL-10 (**G**) and IL-4 (**H**) in spinal cord tissue to β-actin. (**I-J**) Exosomes inhibited Iba-1 expression in spinal cord tissues. (**K-N**) Representative graphs showing the relative production of IL-6 (**K**), IL-1β (**L**), TNF-α (**M**), and INOS (**N**) by ELISA. (**O**) The immunohistochemistry analysis showed that exosomes downregulated TNF-α, IL-1β, and IL-6. Scale bar = 100 μm. *p < 0.05, **p < 0.01, ***p < 0.001

Next, we analyzed the effects of UC-MSC-derived exosomes on inflammation after SCI in rats. First, we found that SCI stimulated tissue Iba-1 activation, while exosomes inhibited it (Fig. 7I-J). The Western blot results showed that SCI upregulated IL-1β, TNF-α, INOS and IL-6, but exosomes significantly inhibited the expression of inflammatory tissue cytokines (Fig. 7A-E). The changes in anti-inflammatory factors are opposite to those in pro-inflammatory factors (Fig. 7F-H). The immunohistochemical analysis and ELISA showed that SCI rats exhibited significantly higher inflammatory cytokines than Sham rats, which was sharply reduced by exosome treatment (Fig. 7O). ELISA results also demonstrated the influence of exosomes on tissue inflammation (Fig. 7K-N).

### Suppression of spinal cord cell apoptosis by UC-MSC-derived exosomes in rats

**Fig. 8:**
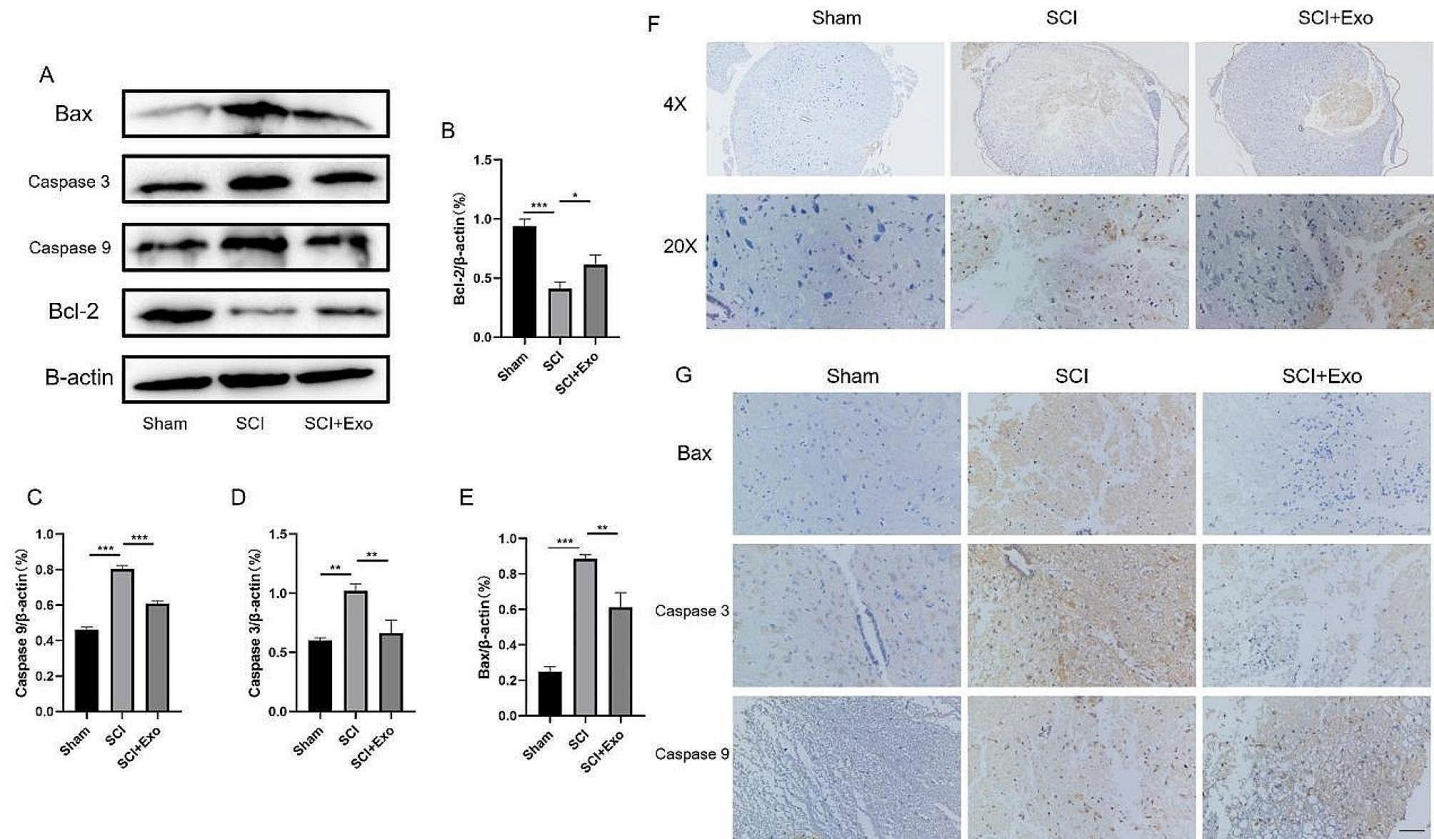
UC-MSC-derived exosomes inhibited the apoptosis of spinal cord tissue in rats. (**A**) Effects of exosomes on the apoptosis of spinal cord tissue by Western blot. (**B**) Bcl-2, (**C**) Caspase 9, (**D**) Caspase 3, and (**E**) Bax levels relative to β-actin in the spinal cord. (**F**) TUNEL staining showed that the number of positive apoptotic cells in the spinal cord tissues of the exosome group was lower than in the SCI group. (**G**) The immunohistochemistry analysis showed that the exosomes inhibited Caspase 9, Caspase 3, and Bax expression. Scale bar = 100 μm. *p < 0.05, **p < 0.01, ***p < 0.001

Subsequently, we explored the effect of UC-MSC-derived exosomes on the apoptosis of rat spinal cord tissues. The Western blot results were consistent with our prediction. Exosomes inhibited the apoptosis of SCI tissues, suppressed Caspase 9, Caspase 3, and Bax, and enhanced BCL-2 (Fig. 8A-E). The TUNEL staining showed that compared to the tissues from the Sham group, the tissues from SCI rats exhibited dramatically elevated cell apoptosis. However, this elevated cell apoptosis was significantly suppressed by exosome administration (Fig. 8F). The immunohistochemistry results showed that the number of apoptotic factor Bax, Caspase 3, and Caspase 9-positive cells was the highest in the SCI group. In contrast, the exosome treatment significantly reduced the number of apoptotic factor-positive cells (Fig. 8G).

### UC-MSC-derived exosome inhibited NF-κB and MAPK activation in the spinal cord of SCI rats

**Fig. 9:**
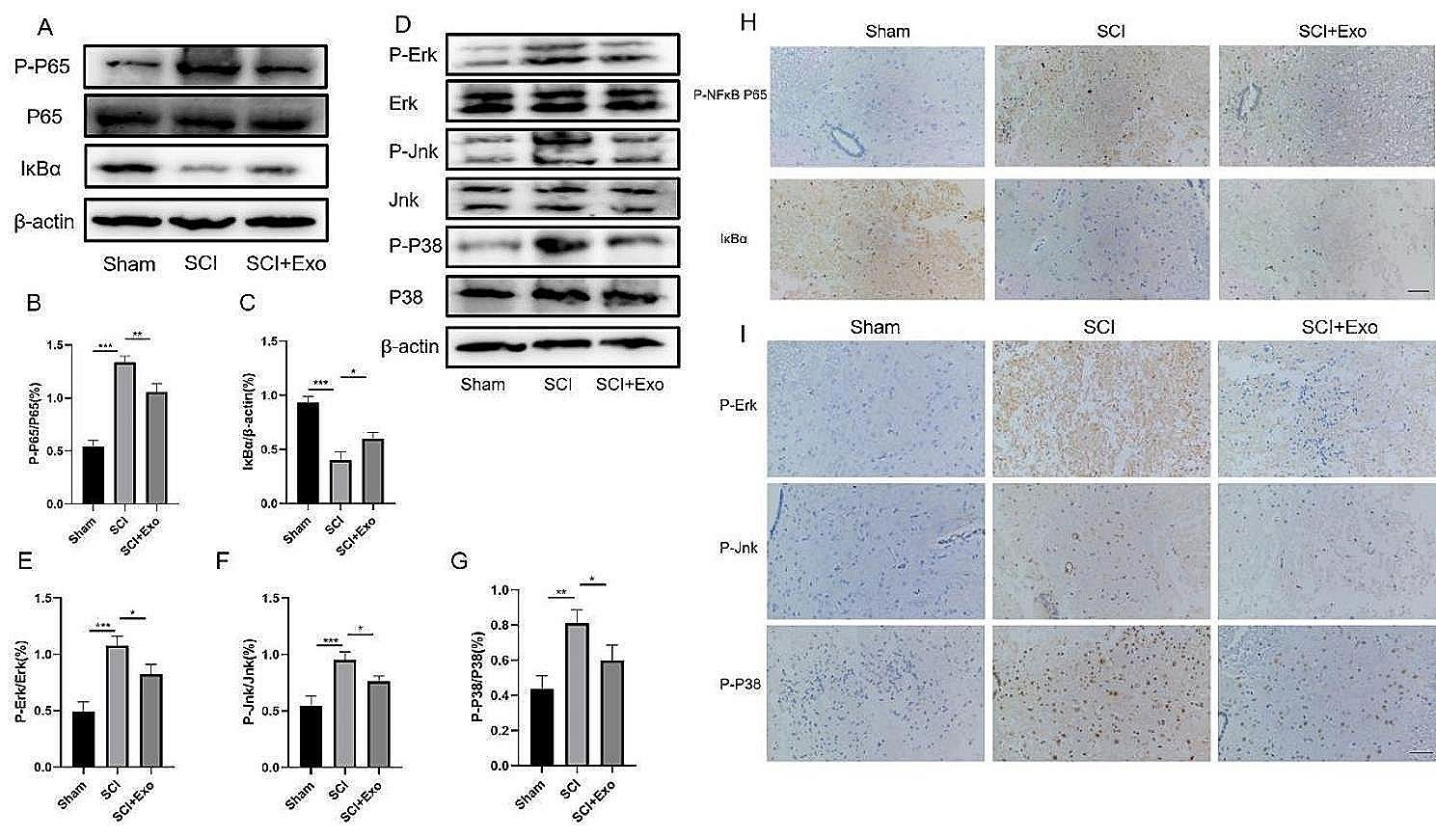
UC-MSCs-derived exosome inhibits the MAPK/NF-κB pathway in the spinal cord. (**A**) The Western blot showed that exosomes downregulated P-P65 in the spinal cord and promoted IκBα expression. The P-P65 to P65 (**B**) ratio of IκBα(**C**) to β-actin. (**D**) Western blot showed that exosomes inhibited P-P38, P-Jnk, and P-Erk expression in the spinal cord tissue. (**E-G**) Representative graphs showing the relative expression of P-Erk (**E**), P-P38 (**F**), and P-Jnk (**G**). (**H**) The immunohistochemistry analysis showed that exosomes inhibited P-P65 expression in the spinal cord and promoted IκBα expression. (**I**) Immunohistochemistry showed that exosomes inhibited P-P38, P-Jnk, and P-Erk expression in spinal cord tissue. Scale bar = 100 μm. *p < 0.05, **p < 0.01, ***p < 0.001

Based on the RNA-seq results, we evaluated the effects of UC-MSC-derived exosomes on the NF-κB and MAPK pathway activation in the spinal cord of SCI rats. Similar to the cell experiments, exosomes inhibited the SCI-induced increase in P-P65 levels and promoted IκBα expression (Fig. 9A-C). The immunohistochemical results also showed that exosomes significantly suppressed the activity of the NF-κB pathway in the spinal cord (Fig. 9H). The Western blot showed that exosomes also impaired P-P38, P-Jnk, and P-Erk levels (Fig. 9D-G). The immunohistochemical results also showed that exosomes inhibited the MAPK pathway in SCI rat spinal cord tissues (Fig. 9I).

## Discussion

SCI causes changes in the function and structure of the spinal cord for various reasons, often resulting in complete or partial mobility loss [27]. Falls, car accidents, falls from heights, fights, and sports injuries can lead to SCI. Moreover, SCI exerts a dual toll on patients, severely impacting their mental and physical health and imposing significant economic stress [28]. The number of new SCI patients worldwide is about 250,000 to 500,000 annually, with more than one million SCI patients in China [2]. The patients themselves have a serious psychological burden but also burden the family and society. One of the most formidable medical challenges is regenerating and repairing SCI. However, there is a lack of effective methods to promote neurological recovery, which can only provide supportive relief for patients with lifelong disability [29].

Herein, we extracted exosomes derived from UC-MSCs and successfully identified them using Western blot, NTA, and other methods. Then, we differently treated BV2 microglia: one group was treated with LPS + ATP and the other with LPS + ATP + exosomes. Microglial cells are the main immune cell type in the parenchyma of the central nervous system (CNS), accounting for 5–10% of the total number of cells [30]. Microglia can guide endothelial cells to influence the formation of blood vessels in the parenchyma [31]. In a healthy CNS, microglia have branched protrusions through which they can dynamically monitor parenchyma to detect infections or injuries rapidly [32]. Microglia mainly have two phenotypes, M1 and M2. Microglia in the M1-polarized state have phagocytic functions and produce pro-inflammatory cytokines and bactericidal molecules [7]. Alternately activated M2 phenotypes are involved in the repair of damaged cells and inflammatory responses in resistant tissues [33]. The microenvironment of tissues is the main factor influencing the polarization state of tissues [34].

Apoptosis is a prominent SCI feature. Due to mechanical trauma and other reasons, some cells in the lesion site become necrotic when SCI occurs, while others undergo apoptosis. Also, evident apoptosis of neurons and oligodendrocytes can be observed in the white matter [35]. By analyzing the apoptosis time of SCI rats, it was found that neuron apoptosis occurred as early as four hours after injury and peaked eight hours later. Apoptosis of glial cells was detected four hours after injury and peaked 24 h later, while apoptosis of oligodendrocytes was detected 24 h after injury and peaked eight days later. A reduction in cell count was also observed up to three weeks after SCI [36]. Therefore, apoptosis of neurons, glial cells, and oligodendrocytes might play an adverse role in SCI. On the one hand, apoptosis during SCI reduces the number of neurons and oligodendrocytes, which cannot meet the needs of nerve regeneration. On the other hand, the apoptotic signals secreted by apoptotic cells, while further inducing the apoptosis of other cells, can cause serious inflammatory reactions, aggravate the pathophysiological SCI microenvironment, and are not conducive to the differentiation of nerve cells and injury repair. Additionally, the inhibition of BV2 microglia apoptosis and SCI tissue by the UC-MSC-derived exosomes was observed by Western blot, immunofluorescence, and immunohistochemistry experiments in vivo and in vitro. In the acute phase of traumatic SCI, a series of physiological changes occur at the injury site, generating a large number of ROS [37]. Due to its multi-origin, persistence, and chain reactions, ROS generated will further enhance inflammatory response at the SCI site, extend the damage to proteins, lipids, and nucleic acids, and induce neuronal necrosis or apoptosis [38]. Here, we found that LPS + ATP promoted cell production of ROS, while UC-MSC-derived exosomes inhibited it, which might be an important reason exosomes promote SCI recovery.

Inflammation plays an important role in SCI. Failure of the blood-spinal barrier and blood vessel rupture caused by SCI leads to spinal tissue bleeding, followed by the invasion of various immunoinflammatory cells in the blood, such as macrophages, T and B lymphocytes, neutrophils, and monocytes into the spinal tissue. The inflammatory cells mentioned above release numerous pro-inflammatory factors in the SCI microenvironment, including TNF-α and IL-6. The serum content of TNF-a in SCI patients increases immediately after injury and with increased injury time, and many TNF-positive cells are detected in the injured spinal cord [39]. The concentrations of IL-6 and IL-1β increase significantly at and around the injury site 3 to 24 h after injury [39]. TNF-α can promote macrophage migration to the injured area and accelerate neuronal death. The increase of IL-1β and IL-6 concentration can cause activation and proliferation of astrocytes and macrophages/microglia, accelerate the formation of connective tissue scar, and aggravate injury severity. The infiltration of immune cells and inflammatory factors further aggravate the inflammatory response of the spinal cord [40]. We used Western blot, immunofluorescence, and immunohistochemistry analyses to demonstrate that UC-MSC-derived exosomes can inhibit the inflammatory response of BV2 microglia and SCI tissue in vivo and in vitro.

The activation of macrophages often accompanies the occurrence of inflammation. LPS can bind to the TLR4 receptor on the macrophage surface to activate downstream Akt and MAPK/NF-κB signaling pathways mediated by this receptor [41]. Hence, inflammatory genes and protein levels are significantly connected to the transcription factor NF-κB and MAPK in cells. Our RNA-seq of BV2 microglia suggested that UC-MSC-derived exosomes might act on the MAPK/NF-κB signaling pathway. Studies have shown that LPS-induced inflammatory responses are mainly mediated by MAPK/NF-κB signaling pathways [42], of which NF-κB is the central link. Through inducing IKK kinase activation, LPS can cause the phosphorylation of IκBα protein and the ubiquitination degradation of the phosphorylated IκBα protein, resulting in the release of NF-κB dimer into the nucleus. MAPK and NF-kB signaling pathways are closely related to the occurrence of inflammation, and they have many overlaps in signal transduction. The MAPK signaling pathway is believed to be the main upstream site for NF-kB activation because 1) MAPK is activated by multi-effect regulatory factors of multiple damage response genes, and the continuous activation of MAPK leads to the continuous production of inflammatory cytokines, activating MAPK and NF-kB signaling pathways, and leading to the cascade “waterfall” effect; (2) after MAPK activation, IkB can be phosphorylated, resulting in IkB degradation and release of p50-p65/RelA heterodimers, which directly lead to NF-kB activation and translocation, driving NF-kB target genes transcription in the nucleus [43, 44]. Therefore, regulation of the NF-κB/MAPK signal transduction pathway plays an important role in controlling the occurrence and development of inflammation. We demonstrated that UC-MSC-derived exosome could inhibit the NF-κB/MAPK signaling pathway in BV2 microglia and SCI rat tissues in vitro and in vivo.

In summary, we found that extracellular vesicles derived from UC-MSCs can alleviate inflammatory responses and promote SCI recovery by inhibiting the NF-κB/MAPK signaling pathway. These findings could offer novel perspectives on future SCI treatments.

## Data Availability

No datasets were generated or analysed during the current study.
